# A Visit to the Hospitals at Montreal

**Published:** 1894-09-08

**Authors:** 


### CANADIAN HOSPITALS.

(By Our Own Correspondent.)

### I.—A VISIT TO THE HOSPITALS OF MONTREAL.

Almost side by side, under the shadow of the wooded mountain from which the city of Montreal takes its name, stand the oldest and the newest of its hospitals, the Hotel Dieu and the Victoria Hospital. Contrasts these are in every way; not merely old and new, but French and English, the one nursed by nuns who have given up the world, the other by bright and energetic women, who feel that the world is before them; the one shut in by high convent walls, the other open to the sunshine and the air.

Nowhere that the flag of England flies, except in Lower Canada, could one find anything like this Hotel Dieu of Montreal. Rather ought I to say the Hotel Dieu of Ville-Marie, for the French population still cling to the name by which they replaced the Indian title of Hochelaga; and as in all the Province of Quebec the French population outnumber the English, their language and ideas still exercise a great, if not a dominant influence. I had learned at Quebec that it is possible to find subjects of the Queen who speak no language but French. I had found in Montreal that every announcement, even to the "Don't walk on the grass" of public gardens, had to be repeated in both languages, and when I went to the Hotel Dieu I had to drag my rusty French from its hiding place before I could obtain an entrance. Yet not even the most Britannic of Britons would grumble that in an institution which owes its being to the courage and devotion of a French woman her language should still be retained.

The foundation of the Hotel Dieu of Montreal is one of the romances of hospital history, and it is noteworthy that though the institution soon passed, as was inevitable under the circumstances, into ecclesiastical hands, the man and the woman to whom it owed its first conception and existence both belonged to the laity. M. de la Dauversière, a gentleman of Anjou, whose austerities rivalled those of the strictest monks, received in a vision the command to institute a hospital on the Isle of Montreal in New France. He was married and had a large family; he had not long before lost the greater part of his fortune, therefore the only way in which he could obey the divine command was to organise an order of nursing sisters. A small and poor convent at La Flèche, of which he and his brother were administrators, formed the nucleus of the order, but still M. de la Dauversière saw no way of transporting them to Montreal, of which he himself knew little, but had learned that the island where the hospital was to be situated belonged to a private individual, M. de Sanson. It appeared, however, that M. de Sanson had received the island on condition that he would colonise it, an order he had failed to obey, and he was easily persuaded to give it up to M. de la Dauversière.

As a first step a company of colonists was formed, skilled in various trades, and all able to fight the Iroquois who would oppose their landing. The Iroquois, however, meant wounds and death, and, while it was not thought advisable to bring the new sisterhood over, one woman was needed "d'un courage héroique et d'une résolution mâle," says her historian, to attend to the colonists' wants and nurse the sick. This woman presented herself in the person of Jeanne Mance.

Jeanne Mance, a young lady who had been from her infancy notable for her piety, had yet felt no vocation for the cloister, then the inevitable home of a religious life; but, hearing of a lady who had established an Ursuline convent at Quebec, she, too, became inspired with an intense desire to go to Canada. Her confessor at first discouraged the plan, but, seeing how fixed the idea was in her mind, he yielded to her wishes, and arranged for her going with M. de la Dauversière's expedition. The girl's resolution created great excitement in France, and ladies of the highest station' including the Queen and Anne of Austria, the queen mother, sought her acquaintance. In reply to their questions, she could only say that she felt that God wished her to go to Canada, but why she could not say. In the spring of 1641 she set out, the only woman among a company of men. The vessel in which she sailed was small, containing only twelve men; they soon lost sight of the two other ships that held the rest of their party, and it was not till August that they arrived at Quebec. There she passed the winter, and went on in the spring to Montreal. Meanwhile the excitement which her bold step had caused in France resulted in gifts of money, more than was necessary for the building of such a hospital as was then required. Amid endless anxieties and dangers the hospital was built, the workmen labouring, like those whom Ezra employed to build the Temple, with the sword ever at their side. It was of wood, as were all Canadian houses then, and many even now, and was situated above the fort, but not far from the river, in what was afterwards known as St. Paul Street. This building was finished in October, 1644.

New colonists now began to come over, and Mademoiselle Mance had a few women companions; but between the attacks of the Iroquois and the indifference of friends at home, the colony was nearly destroyed. Mademoiselle Mance returned to France on a visit, and succeeded in rousing the flagging zeal of their supporters. But the Iroquois attacks continued. At one time the hospital became no longer safe to dwell in, for in the garrison at Montreal there were only seventeen men able to bear arms, and none could be spared to protect it. But the colony survived all its troubles, and grew till in 1659 Jeanne Mance thought it advisable to bring over those nursing sisters of La Fleche who during these years had been training themselves in France for this, which they regarded as their destined work. The trouble and dangers of the institution were by no means past. Labour, privation, and danger were the portion of the sisters for long years to come. Fresh fish and meat were luxuries to be religiously kept for the patients. The sisters lived on salt fish and such vegetables as they could raise in their little garden behind the walls, thick as a fortress, of the hospital. Fruit grew in the neighbouring woods, but they dared not gather it for fear of the stealthy and cruel Iroquois. In winter the water and even the patients' wine froze on the table. And yet they laboured on, sustained by faith in God and love for humanity. And among their patients some endowed them with little farms and strips of land in the neighbourhood, which since Montreal became a great city, has made the once starving community powerful and rich.

